# Supplementary material for: Dataset search in biodiversity research: Do metadata in data repositories reflect scholarly information needs?
Source: PLoS One. 2021 Mar 24;16(3):e0246099. doi: 10.1371/journal.pone.0246099 (PMC7990268; doi:10.1371/journal.pone.0246099)
Supplement: Attachment — Submitted filename: response_to_reviewers.pdf [file pone.0246099.s001.pdf]

FRIEDRICH-SCHILLER-UNIVERSITÄT JENA

Department of Mathematics and Computer Science

Friedrich Schiller University Jena · Institute of Computer Science · 07743 Jena · Germany

Felicitas Löffler
Heinz Nixdorf Chair for Distributed Information Systems

Ernst-Abbe-Platz 1-4
07743 Jena

Germany

Telefon: 0049 (0) 36 41 9-46413
Telefax: 0049 (0) 36 41 9-46302
E-Mail: felicitas.loeffler@uni-jena.de

PlosONE Editorial Team

Jena, 5. December 2019

Dear Robert Guralnick,

We thank you for your kind comments and the reviewers for their thorough feedback on our manuscript following our submission for publication.

In accordance to the addressed issues, we have revised the manuscript. Please find attached to this letter a detailed reply on all issues. All line numbers refer to the manuscript with track changes.

We look forward to hearing from you regarding our submission. We would be glad to respond to any further questions and comments that you may have.

Kind regards,

Felicitas Löffler

PhD student and Research Associate

Friedrich Schiller University Jena, Germany

FRIEDRICH-SCHILLER-UNIVERSITÄT JENA

Issues both reviewers have mentioned

grammatical errors	We agree with the referees, and therefore we asked colleagues possessing fluent English knowledge to proofread the paper.
cutting the length, focusing on key points	We also agree here. We have cut the paper by 6 pages. For instance, we left out the annotation guidelines in Section A and shortened the results in Section C. Now, the manuscript.pdf file contains 33 pages.

Reviewer 1

I suggest changing the title, as it is not quite grammatically correct, and "findable" is of course only one component of FAIR. Having FAIR in the title implies that you would some way address all aspects of FAIR.	The reviewer is right to point out that we did not address all FAIR aspects, thus, we changed the title accordingly.
The first paragraph of the abstract discusses data reuse in general, but this is not what the paper is about. This paragraph should be revised to be specifically relevant to the paper. I suggest in this revision to ensure that the first sentence is eye catching and relevant. "For a number of reasons," is not a great start for an abstract. The abstract in general is too vague. You should briefly indicate the general method for determining search interests here.	In accordance with the referees' wishes, we have now changed this paragraph in the abstract.
DataONE is working on quantifying FAIR in datasets and in repositories. They have found that Findability and Accessibility are covered reasonably well, but interoperability and reusability are not at all. You might look into what they are doing. At least address the fact that while Findability is still an issue, we are doing better here than in other aspects of FAIR.	We accept this hint from the reviewer, and we added this initiative to the related work section (see Lines 387 - 391).
You have to consider the scope of a particular repository to determine what is feasible to use as a metadata standard. A generalist repository cannot serve specific search interests for each domain represented. Therefore much of the responsibility of making data findable rests on the scientists describing their data. There are also opportunities for this are in general fields: the title, abstract, keywords,	It is true that primarily scientists are responsible for proper data descriptions to make their data findable, but data repositories could offer curation services to guide scholars in the data submission process and to encourage them to describe their data thoroughly. Hence, we added a paragraph addressing this issue in Section D, Subsection "Challenges" (Lines 1155 - 1163)

and methods, where they can ensure that relevant terms are included.	
Line 77: Others are working on evaluating FAIR, and Findability happens to be the most well-covered thus far. There is definitely a lot of work to be done here, but is this statement is accurate?	We agree that a lot of work towards FAIR and findable data has been made in recent years, but Information Retrieval algorithms that are used in existing search engines were mainly developed for text. The specific needs for dataset retrieval have not been analyzed entirely. The results from previous user studies in data portals confirm our assessment (see Section Related Work). Therefore, we think that the statement “dataset retrieval is a yet unexplored research field” is right and have clarified this focus on datasets in Line 80.
"Authors refer to “biodiversity” as a field. This should be “biodiversity research/science” or “biodiversity informatics.”"	We agree with the referee. Thus, we have changed the term accordingly throughout the paper.
Much of the Related Work section does not seem focused on the research questions (which are also unclear as mentioned previously). The paper should focus more on what the authors actually did, and less on specific methods done in past studies. For example, you should discuss how search works in the specific repositories where you evaluate metadata.	It is true that the research question was not properly addressed. We changed the introduction based on the reviewer’s comments.<br><br>We also added a paragraph in the related work section, which lists the search engines used at the repositories we evaluated (see Lines 331 – 339).
169 questions generated by 73 scientists does not seem like a sufficient dataset for broad search needs in biodiversity research. Although, the general categories of information produced seem reasonable.	We understand the reviewer’s concerns. Therefore, we addressed this issue in Lines 445 – 451.
In recommendations to data repositories in Table 10: What do you mean by #4? For most repositories, data providers are responsible for “filling in” metadata elements and providing descriptive metadata. And, it is not possible to do this when serving a broad community.<br><br>"Lines 1279-1284: Many fields may not be relevant for a specific dataset, so filling in all metadata fields is not a realistic recommendation	We agree with the referee that this recommendation was not well formulated. We changed this statement in the recommendation box and the related paragraph (see Table 12 and Lines 1121 – 1122).
I do not understand how you determined whether metadata provided in the repositories was rich? Is it only by whether certain fields have associated metadata provided? You can have rich metadata descriptions in general fields, title/abstract/methods with terms that address the topic areas you found to	We also agree concerning the methodology of the content analysis. Hence, we added a more thoroughly content analysis on general fields. In October and November 2019, we downloaded all available title, description and keyword fields from all repositories in OAI-DC metadata standard. In addition, we collected,

be important. Particularly for generalist repositories, you cannot have an abundance of domain-specific metadata elements. To evaluate metadata richness for findability, I think that a better approach would be to look at the actual terms provided in the general metadata elements (e.g. title/keywords/abstract). The content analysis mentioned for specific repositories seems somewhat haphazard. Did you have a specific approach for this?	counted and sorted the keywords by frequency. The methodology is presented in Section C, Subsection Methodology (Lines 849 – 872). The results are described in Section C, Subsection "Results" (Lines 935 – 982).

Reviewer 2

"The main objection is that the paper is constructed around the idea of the direct correspondence between findability and metadata richness. While this correspondence exists, it is not the only FAIR factor in the specific case of biodiversity research."	Reviewer 1 mentioned similar concerns about the correspondence to the FAIR principles. Therefore, we have completely revised the introduction.
GBIF - Special case in search<br><br>"in the specific case of biodiversity, a significant amount of searching occurs directly on the repository contents" e.g., GBIF: "Searches in GBIF are done through structured querying within the index and therefore can easily bypass the metadata descriptors"<br><br>"the description of the study and its results seem confusing or shallow when applied to the specific case of retrievability of biodiversity data. A clearer examination of the retrieval mechanisms; a discussion of the particularities of biodiversity data repositories, and a comparison to other completeness studies, seem necessary."<br><br>1125 The statement is highly debatable. Search engines tailored to contents (such as e.g. GBIF) are very highly specific (albeit also highly structured) and often do not require metadata search at all in order to produce and retrieve resultsets.<br><br>1182-1194 needs rewriting. Also, the main idea is incomplete. As I said before, certain repositories e.g. GBIF are aimed at producing resultsets from queries redirected to their contents and can bypass metadata search entirely. It is difficult to apply the results of metadata completeness to such cases. Data completeness has been studied and published; for example, Gaiji et al. (2013) (DOI:	The referee is right to point out that in GBIF's case occurrence data records are directly indexed in Darwin Core format. However, the OAI-PMH interface does not allow a download of the occurrence records but only offers the metadata of the belonging datasets. We clarified that in the introduction (Lines 64 – 65) and the methodology of Section C (Lines 804 – 808), and we also considered that in our discussion in Section D (Research Contributions, Data Repositories) (Lines 1039 – 1042).<br><br>We also extended the content analysis of the repositories evaluated. The results demonstrate that biodiversity data are contained in domain-specific archives as well as generalist data repositories. (Lines 935 – 982)

10.17161/bi.v8i2.4124) and Otegui et al. (2013) (DOI: 10.1371/journal.pone.0055144) in the case of GBIF. It would be highly desirable to discuss this difference and explore the interplay between these two approaches.	
81 I believe this is "dataset retrieval". A dataset search can hardly be characterized as an application—it is a procedure, that may use an application.	We agree with the reviewer, and we changed the naming to dataset retrieval throughout the paper.
113-114 There is literature already available about user needs in the realm of biodiversity data.	In accordance with the referees' hint, we added that user study to the related work (Line 271 – 279) and compared their outcome with our result of the search interests (Section A, Subsection Summary) (Lines 657 – 661).
123 This definition of metadata richness seems a bit restrictive. Metadata describe the data, and therefore can be rich if the description is as complete as possible. This completeness helps ensuring search success, but search is not the only reason for metadata. Rich metadata may also help classifying datasets or deriving knowledge from the metadata, rather than from the metadata they represent.	It is true that this definition was a bit restrictive. Therefore, we revised this definition.
156 I disagree in that a Research Question must be broad and semantically complex. There are research questions which may be formulated in very narrow terms and perhaps be directly answered by simple searches. For example, this very paper addresses whether metadata are rich enough for efficient search in biodiversity. An associated research question would be what the user needs are when they query biodiversity databases: in fact, an answer to this question is required to properly formulate the other question, for searches in biodiversity are driven by what the users' needs for information or knowledge are. That research question (user needs) might, and has been, solved by a meta-analysis of literature where the data used were in fact literature metadata using a single query (Ariño et al., 2018: DOI:10.3897/biss.2.25738) where keywords are tallied, and by a survey (Faith et al., 2013: DOI:10.17161/bi.v8i2.4126) using highly focused questions addressed to a dataset of practitioners. While the authors may be tempted to dismiss these as "search questions", they are also final goals in themselves (thence, "research questions"). In my view, the distinction that authors make between Research Questions and Search Questions is not correctly	The referee is right to point out that a direct distinction between search and research questions can not be drawn. We removed that part from the paragraph.

formulated. It seems as if the authors actually wanted to make the distinction as having metadata as a gateway to answer research questions, while search questions would be answered by the data described by the metadata (but not by the metadata), but not explicitly stated as such. At any rate, I don't think the distinction applies.	
270- The RDA DDG study mentioned here concerns itself with general search systems but this paper focus on biodiversity data repositories. The community of users tends to use a fundamentally different approach where the search granularity afforded by specific engines grants precise resultsets based not on keywords but on actual contents. The argument that "there is a need to analyze the given metadata and to quantify the gap between a scholar's actual search interests and the underling metadata" is valid for the general case, but perhaps less so for the specific case of biodiversity data. A deeper and more tailored argument, with examples, might be required here.	We understand the reviewer's concerns. We suppose, the referee assumes that biodiversity data is only available in specific repositories. Our content analysis reveals that this not the case (Lines 935 – 982). Biodiversity data are also available in generalist data repositories. Hence, metadata descriptions are necessary to find relevant data.<br><br>The RDA study did not publish the participating repositories. However, at least the covered domains, being potentially relevant for biodiversity research, are mentioned (see Line 7 – 10).
357 I don't know about such question corpus aimed at metadata, but I also very much doubt that a corpus of questions aimed at retrieving specific datasets does not exist. For example, GBIF stores al queries resulting in resultsets and caches them for later, more expedite answer if the question is repeated and the underlying data have not changed. This, by definition, should constitute a question corpus—but I do not know whether the corpus, as such, is public or available on demand. I believe that the statement in lines 357/358 should more properly phrased as "there is no published log analysis of a question corpus for the biodiversity domain".	We agree with the reviewer that this statement was too general. We revised it accordingly to "there is no published log analysis" (Line 325).
466-477 The summary of the methodology is rather unclear and remains so until the detailed description has been read. Either write (or, better, plot) a clearer summary, or do away with it.	We agree with the reviewer. We removed the summary of the methodology and just added an introductory sentence (Lines 441 – 442).
Figures:	
p. 46, Fig. 1: the color scheme is not clear at first sight. Intended to mean: blue, backend: brown: user interface. Shadowing the BACKEND and USER INTERFACE titles would help. Footer (p. 6) says Green for crawling but no green is visible in the figure.	It is true that there was a mistake in the caption. We corrected that (Figure 1). The indexing process, which runs frequently in the background, is colored in blue. The retrieval process (orange) does not only cover the user query and the returned ranked list but also

	comprises the actual search, which takes place in the backend, too.
[Figures 3a&b could be combined. Figures 4a&b could be combined. Figures 5a&b could be combined. Why red bars?	In accordance with the reviewers suggestions, we combined the figures (see Figure 3, Figure 4a and 4b)
Figure 7a, 8a, 9a, 10a, 11a: why not cumulative? Trends are much more visible and easier to compare. Also, data for 2019 is irrelevant: the year is not over yet.	We agree with the reviewer's suggestion that cumulative timelines would be more visible. Unfortunately, the data are too large to process them all at once, but we computed the timelines again and omitted data from 2019.
Figure 7b, 8b, 9b, 10b, 11b: Apparently the Y axis is not percent but fraction. Difficult to read. Suggest vertical format. What is the ordination criterion? Why not ordered by %?	In accordance with the referee's proposal, we present the metadata field usage in revised figures (see Figure 7a – 7e).
