# Supplementary material for: Dataset search in biodiversity research: Do metadata in data repositories reflect scholarly information needs?
Source: PLoS One. 2021 Mar 24;16(3):e0246099. doi: 10.1371/journal.pone.0246099 (PMC7990268; doi:10.1371/journal.pone.0246099)
Supplement: Attachment — Submitted filename: response_to_reviewers.pdf [file pone.0246099.s002.pdf]

FRIEDRICH-SCHILLER-UNIVERSITÄT JENA

Department of Mathematics and Computer Science

Friedrich Schiller University Jena · Institute of Computer Science · 07743 Jena · Germany

Felicitas Löffler
Heinz Nixdorf Chair for Distributed Information Systems

Ernst-Abbe-Platz 1-4
07743 Jena

Germany

Telefon: 0049 (0) 36 41 9-46413
Telefax: 0049 (0) 36 41 9-46302
E-Mail: felicitas.loeffler@uni-jena.de

PlosONE Editorial Team

Jena, 14. May 2020

Dear Hussein Suleman,

We thank you for your kind comments and the reviewers for their thorough and constructive feedback on our manuscript following our submission for publication.

In accordance to the addressed issues, we have revised the manuscript. Please find attached to this letter a detailed reply on all issues. All line numbers in the right column refer to the manuscript with track changes. To facilitate the review, we highlighted new sentences in red color. Revised paragraphs or paragraphs moved and revised are highlighted in blue color. Paragraphs that were moved without any change in content are highlighted in green.

We look forward to hearing from you regarding our submission. We would be glad to respond to any further questions and comments that you may have.

Kind regards,

Felicitas Löffler
PhD student and Research Associate
Friedrich Schiller University Jena, Germany

Issues both reviewers have mentioned

Typos and grammatical errors, unclear sentences	In accordance with the reviewers' suggestions, we have corrected the typographical and grammatical errors.

Since Reviewer 2 raised no additional concerns, in the table below, only comments by Reviewer 1 are reflected

Background section<br><br>"the background information can be cut"<br><br>Line 150: The intro to background is a bit unclear. Do you mean: "This section provides background information on a variety of inputs involved in a search process…"?<br><br>Is detailed background on the retrieval process necessary? I think that brief information on the exact retrieval process used in the repositories analyzed would be more useful for this paper. And focus on the situation with dataset retrieval and searches focused on standard metadata.	 <br>We agree that not all parts of the background section are relevant for further reading. Therefore, we removed the background section completely and only described the necessary background information at the beginning of the 'Dataset search' paragraph in the Related Work Section (Lines 248-280).
Related Work<br><br>There is also too much detail in the related work section. You can greatly reduce the amount of background information provided by focusing specifically on background information directly related to this work. I suggest some brief statement (a couple of sentences or maybe paragraph) about work in other fields followed by specific information that is more directly relevant. The GBIF portion is most relevant. The interesting thing here is that we can use methods developed and more thoroughly explored in other fields to answer questions related to biodiversity dataset publication and use. You do not need the details of results for those other studies.	 <br>In accordance with the referee's wishes, we have now shortened the 'User Interest' part (Line 173-188) as well as the 'Dataset Search' paragraph in the Related Work Section (Lines 248-280).
Objectives<br><br>The objectives section is redundant with the objectives already presented in introduction? I think you can cut out multiple pages here still. Maybe even completely cut or move to supplement the entire Retrieval Process, Related Work, Dataset Search,	 <br>We accept this hint from the reviewer, and we moved some sentences to the introduction (Lines 41-56) and omitted the Objectives section.

Objectives section. You could summarize all of this into a couple of paragraphs in the main intro.	
Question Analysis<br><br>Line 470: why is EASE identified here but not discussed in the standards section? I am not familiar with this.<br><br>Line 478: I do not see a good description of the Quality category. Do you have a better description of this that you provided to annotators?<br><br>Lines 537-539: The results section includes a mix of methods and results, so consider renaming this section.	It is true that EASE is not further considered in the Metadata Schema Section. Our analysis is based on the filtered result list in re3data. We mentioned and described the filtering in the text. In addition, we added a hint to the caption of Table 2. We also extended the description of EASE in the Related Work Section (Lines 295-299).<br><br>The referee is right to point out that the description of the Quality category is inappropriate. We extended the description accordingly (Line 387-389).<br><br>We understand the referee's concerns. Due to partial explanations of the metrics used, we renamed the Section to 'Results and Metrics' (Line 450).
Metadata Schemes<br><br>For the annotations of entities from the researcher questions, how do you map the entities/concepts that you identified to metadata fields from existing standards?	The referee is right to indicate that we did not provide the concrete mapping of metadata fields and categories. We added the Excel file with the individual mappings to our GitHub repository and mention this in the manuscript (Lines 638-639).
Metadata Usage<br><br>(Line 880): In general, the metadata usage section I think is really important, but the writing is a little hard to follow and not as well organized. You need to reorganize this section to clarify key points. I really like how you are identifying what metadata elements are identified in general fields like keywords, versus more specific metadata elements, but this is not well explained and hard to follow.<br><br>Line 904: Why would you filter using a group of relevant keywords for Dryad, and only "Biodiversity" for Zenodo and Figshare? This does not make sense to me.	We agree with the referee that the structure of the Metadata Usage Section was hard to follow. Therefore, we added introductory sentences explaining the key points and revised the paragraph headlines in the Methodology subsection (Lines 674-696) and Result subsection (Lines 778-790). We also shortened the paragraph Data Collection and omitted the information about the OAI-PMH interface. In return, we added the URL to the protocol's website (Lines 706-713).<br><br>It is true that this statement can easily be misunderstood. We added a further sentence to clarify our rationale behind that choice (Lines 867-871).

Figshare has a bias towards collection data?	The referee is right to point out that this sentence is not clear. We changed the description in the text accordingly (Lines 878-879).
Discussion<br><br>Lines 940-941: I think you should remove or revise this. It is a real problem if the comprehensibility of your categories is only “fair”. However, Quality seems like the only one that is not well defined and less intuitive. The others match fairly well with metadata elements from existing standards. I also think that you need to better define these categories up front.<br><br>"and some components integrated into the discussion to place results in the context of previous work."<br><br>The discussion should usually present results in the context of previous work, but currently only summarizing your results. You need to revise the discussion to concisely present your results in the context of other work done in this arena. Maybe taking some of the information presented in the background section. However, caution to balance providing some useful and specific context without going into too much detail (as is the case currently in the background section).<br><br>Lines 970-971: What do you mean by this - “repositories did not fully use all provided elements.” And “Most repositories seem to be aware of that problem and enhance metadata with numerous keywords in generic fields such as dc:subject.” The responsibility is usually on the dataset authors to provide this information. Maybe more detail should be required by the repository, and of course it would be nice if there were more of a review process for datasets, but the resources/incentives are not currently there. So, unless I misunderstand the meaning, the current language is not accurate.<br><br>Line 975: clarify sentence, what supports improved filtering in search?	We agree with the referee, and we removed this sentence (Lines 922)<br><br>We agree with the referee that the discussion part needs revision and should include a discussion with respect to related work. We therefore restructured this subsection and added a discussion in the context of previous work (Lines 891-962).<br><br>We understand the reviewer’s concerns and addressed these topics in the discussion (Lines 951-962) and specified the recommendations for data repositories (Lines 1002-1006).<br><br>We agree with the referee that faceted search needs to be better explained. Thus, we introduced this search function at the beginning of the Related Work section (Lines 270-279) and refer to this topic again in the discussion (Lines 951-052).

Recommendations<br><br>"The other major area for improvement is to better incorporate some specific results from your content analysis of search questions into the recommendations."<br><br>Table 12 Recommendations: I don't understand how #4 is for repositories. Should this not be for authors? Repositories are not responsible for describing the data, but they are responsible for providing good user interfaces and options for controlled vocabularies and standards. Also, these recommendations are too general in some cases and should incorporate some of the specific metadata recommendations identified to improve findability for biodiversity research questions.<br><br>Line 1084: remove this sentence, doesn't make full sense. You might at least mention incentives for scholars to provide rich metadata (there are a lot of related issues around publishers not reporting data citations, data citations metrics behind paywalls, not sufficient resources for repositories to review datasets thoroughly etc.).	It is true that the results from the question analysis could be better incorporated into the recommendations. Hence, we addressed this issue in Lines 976-985, Lines 993-994 and Lines 1047-1051.<br><br>We understand the referee's concerns. Hence, we added a sentence to the recommendations for data repositories concerning data curation services (Line Lines 1002-1005) and we extended the respective part for scholars (Line 1046-1051).<br><br>We agree that this sentence can be removed (Line 1086). The rest of this former paragraph was moved to the paragraph before the subsection '*Suggestions to Bridge the Gap*' (Line 955-962)
Figures<br><br>Figures: The titles for axes and legend items should be clarified and standardized across figures to improve presentation and clarity. Figures 6b-e have numerous items in legend, but for most of these it is not clear that all of these are present in the actual figure as presented	The referee is right to point out that the Figures 6b-e have numerous items in the legend but there are only a few timelines present. We clarified these overlapping timelines with an additional sentence in the caption of Figure 5. In addition, we have also adjusted the icons and colors in the charts to ensure that a metadata standard is represented with the same icon and color in all repository charts where the schema is used.
Table captions<br><br>Table 4 - The categories are sorted by frequency of what? Clarify the title<br><br>Table 8 – Again, frequency is not explained in the title.	We agree that the titles could be clearer. Thus, we changed them accordingly (Table 4 and Table 8).
