# Supplementary material for: Dataset search in biodiversity research: Do metadata in data repositories reflect scholarly information needs?
Source: PLoS One. 2021 Mar 24;16(3):e0246099. doi: 10.1371/journal.pone.0246099 (PMC7990268; doi:10.1371/journal.pone.0246099)
Supplement: Attachment — Submitted filename: PONE-D-19-18843R2 Review.pdf [file pone.0246099.s003.pdf]

PONE-D-19-18843R2 Review

Introduction - Main Claims of Paper

The authors state that biodiversity data is hard to find because

1. it exists either across a number of different domain-specific repositories *or* in generalist repositories,
2. there is a disconnect between the search choices made by researchers looking for data and the search methodologies provided by repositories, and
3. even when domain-specific standards include a majority of required fields, many repositories (including domain-independent general purpose repositories) don't make use of these standards.

Therefore, repositories and researchers need to work together to improve findability (the F in FAIR) of the data. The authors make an important point about findability that I have not yet seen published, stating that even if a repository uses richly-annotated metadata formats to describe their data, if the metadata fields included in these formats do not match researchers' search interests, the data will simply not be found.

The authors use the knowledge of biodiversity experts to assign categories to a corpora of facts, and then compare which formats (and therefore the repositories that utilize those formats) align with those categories. Their analysis suggests several major differences between how community experts describe their data and how standards utilized by repositories describe the same data. They use the results of this alignment and analysis to suggest guidelines (also called "checklists" in the Conclusion of the paper, line 1105) for repositories and for researchers so that what researchers search for and what repositories index will be more closely matched. The authors argue that the biodiversity community requires improved findability of data via metadata that is targeted to researchers' search interests. Such metadata will improve searches/findability as well as increase the completeness of dataset curation. The authors state that data standardization is still at an early stage for biodiversity data, and therefore their suggested guidelines are significant for their community. In future, they also suggest that NLP or similar methodologies might help researchers add metadata to their database submission, thus improving completeness of the metadata.

The authors approached the issue by comparing users' searches to what databases provide, which is novel and interesting; I enjoyed learning in what ways users' search terms and repositories' search fields / metadata format fields are different - and that there is a disconnect between the two, especially for generalist repositories that tend to use generalist formats (and not domain-specific formats). As a reader, this research question is highly engaging; the authors have piqued my interest in how researchers' interests and repositories' metadata structures diverge, and where they are the same. Additionally, I thought that the information about the level of completeness of metadata (e.g. around line

811) was interesting, and the authors could have even spent a little more time on discussing the level of completeness of metadata across all repositories.

The comparisons performed between the experts' categorizations and metadata standards used by selected repositories was relevant and different to what I've read about before. Although I am not a statistician, the analyses the authors chose seem suitable and fully support their claim that there is a disconnect between what biodiversity domain experts are searching for and what metadata fields repositories are providing. However, there are two points that require further support (or modification) in the manuscript:

1. Their initial pool of standards and databases is too small, and
2. The guidelines are short and too generic (see Table 12), with the conclusions presented in them being too high-level and less directed at their community (biodiversity) than I would expect of a life science guideline.

The next section of the review discusses these points in detail, followed by a section outlining clarity/grammar suggestions.

Discussion of Issues

The sections below detail the main areas of concerns introduced above, which may prompt a re-analysis of some portions of the data. In particular, if more metadata standards are included in the analysis, then some of the results may need to be rerun. Equally, if the authors accept my opinion on the guidelines in Table 12, a shifting of focus of that section of the manuscript may be required.

Metadata standards

I have two main concerns for the authors regarding the metadata standards:

1. the inexact terminology used to explain metadata standards, and
2. the low number of metadata standards / databases chosen.

Usage of *metadata, schemes, standard,* and *formats*

The way in which the authors define metadata, formats and standards contains some errors, and their discussion of the standards they do discuss is limited. In the paragraph beginning at line 583, there are a few mistakes in how metadata is defined and discussed. In Line 584, they state that metadata describe data in a structured format, and that metadata possess a "schema" that is typically an XSD. Firstly, metadata are *not* schemas; it is the information itself, agnostic of a specific format. Metadata *formats* specify how the metadata should be described, which might be XML via an XSD, but could equally well be one of a multitude of other formats. Such *formats* may ultimately become *standards.* The terminology is very important, and the way this paragraph is currently written is confusing. This continues throughout the manuscript and needs to be normalized and fixed throughout. Another example is the legend for Table 5: "Metadata schemes offered by selected data

repositories". By using the word "scheme" which is very similar to "schema", the authors are continuing this naming confusion. The things listed in Table 5 are *formats*, and more than that they are also *standard formats,* but they are not *schemes.* An additional issue with Table 5 is the inclusion of "RDF" in the table. RDF is not a metadata format, any more than HTML or CSV or XML is. It is certainly an exchange format, a way to store data in a structured way. But it wasn't created purely for metadata, but for any type of data.

Secondly, in lines 588-90, the authors state that in order to become a metadata standard, a schema **needs** to be formally adopted by a standards organization. This is not true; while a standards organization is one route to becoming a formalized standard, most commonly in the life sciences a *format* (not a schema) becomes a recognized *standard* when it is validated by its user community. Indeed, the majority of biological standards have been both created and later adopted by their communities.

Numbers of and selection methods for the standards used

The authors have not fully surveyed the current standards landscape for their domain. I am aware of a number of standards and databases which include biodiversity within their remit which are not presented in this paper, and I would be interested to hear from the authors as to why they were not included.

In the paragraph beginning at line 591 in *Section B - Metadata Standards in the Life Sciences*, the authors state that the standards chosen for evaluation are taken from a search of re3data using the search term "Life Sciences". In this way they discovered 25 standards, 13 of which were suitable for further study. Their methodology for filtering from 25 to 13 standards is sensible, but the number of initial standards in the set (25) is low. An initial search of FAIRsharing for *Life Science* formats with a "ready" status (the equivalent search to that presented in the manuscript) returns 226 standards. A similar search with *biodiversity* + ready + formats returns 10 records, only 1 of which is included in Table 2 of the manuscript. Table 2 includes general-purpose standards (e.g. Dublin Core) and geospatial standards (e.g. FDGC) that are also only a small number of available standards (e.g. there are 34 standards related to Earth Science in FAIRsharing). Comparing 13 standards is simply too small of a comparison when the number of available standards according to their own criteria is so high.

Unless there is a clear reason as to why they are unsuitable for inclusion, an evaluation of a larger pool of standards, such as those suggested here, would be a required addition to the manuscript in order to determine if there truly is a large difference between researchers' search interests and the metadata fields currently available to the biodiversity community in existing repositories.

The authors should either incorporate the additional standards described above within their evaluation or provide reasons why they should not be incorporated.

Guidelines

Based on the information presented in the paper (and presuming that the addition of more standards as described above does not change the conclusions), I would agree that the work they have performed on the disconnect between users and repositories is relevant and showcases a real stumbling block to data discovery in biodiversity. However, the guidelines they describe in this paper are too high-level to be significant for the biodiversity community. I recommend reworking the “guidelines” into something more akin to a “call to the community” to align researchers’ search needs with repositories’ metadata fields. If the authors choose to retain these guidelines in Table 12, they must convincingly argue how they meet the definition of “guideline” as most commonly used in the Life Sciences (their target audience). The next two sections cover 1) the issues with calling Table 12 “guidelines” for the biodiversity community within the context of the life sciences, and 2) a point-by-point discussion of those guidelines, if the authors chose to keep them as they are.

Interpretation of “Guidelines” in the Life Sciences

Table 12 presents the authors’ novel work amongst a number of other points that are very high level, almost hiding the interesting and relevant work discussed in the manuscript (analysing researchers’ search interests against repositories’ search fields). A lot of work has happened recently on quantifying FAIR, making data FAIR and measuring FAIRness. Many of the suggestions here (extending existing standards, using controlled vocabularies, assigning unique identifiers, use of appropriate standards in your field of research, etc) have already been published. Instead, consider repackaging these “guidelines” in Table 12, for two reasons:

1. Focus the readers’ attention on the work presented in the manuscript, and rephrase it as a "starting point for discussion" with the community, or a “call to the community”. Drop the majority of the high-level points from Table 12, instead referencing publications such as https://doi.org/10.1371/journal.pcbi.1005097 for repositories and https://doi.org/10.1371/journal.pcbi.1004525 for researchers. Indeed, the work described in this manuscript would have direct relevance on Findability as per the RDA’s FAIR Data Maturity Model: specification and guidelines - draft, Section 3.2 part RDA-F4-01M, which states that “Metadata is offered in such a way that it can be harvested and indexed”; improving the alignment of researchers’ search interests (the “harvesting” part) and repositories’ search indexes/fields (the “indexing” part) would fit squarely within this section. This manuscript could be used as a jumping-off point to begin a discussion with generalist repositories, or perhaps a sound argument for encouraging researchers to submit to domain-specific repositories that can better serve their data (and better deal with their search interests)? The authors have already made the very good point that biodiversity researchers find it hard to search/submit data to domain-specific repo because their data is deposited/comes from multiple areas. This manuscript seems like an ideal start to a discussion of exactly how existing domain-specific repositories could be more responsive to biodiversity researchers’ needs.

2. The word "guidelines" is loaded with a very particular meaning in the Life Sciences, whether justified or not, and what has been presented in this manuscript does not fit that meaning. When a life science community uses the term “guidelines” to describe a document, the results are a highly structured set of detailed recommendations focused on providing researchers within that community a real guide to how they should treat their data [MIAPE, MIAPE-GE, MIAME, and MIGKD/GMISR among many others]. As a minor but related point, the guidelines are called a “checklist” in the conclusions (line 1105), but they do not fit that definition and that word should be avoided.

Point by Point

This section covers each item in the guidelines, point by point. As discussed above, it may be best to retarget these guidelines by focusing on the points supported by the manuscript. However, if the guidelines do stay in their present form, the authors may wish to consider the following.

DR1

Point 1 for repositories in Table 12 is asking (primarily) generalist repositories to offer domain-specific standards, but this point is offered without any guidance. Around Line 961 and the DR1 item itself: “We are aware that it would require high efforts to introduce more domain-specific metadata schemes at generalist repositories; however, it would improve data descriptions.” Asking generalist repositories to subscribe to what might be 100s of domain-specific standards might not be practical, but the authors have not mentioned this potential stumbling block, other than saying it would require “high efforts”, which may be an understatement. Generalist repositories allow all kinds of research data (Physics, Social Sciences, Astronomy, History, Biology), and some comment by the authors as to the seriousness of this request would be useful.

DR2-5

This is more achievable by generalist and domain-specific repositories alike. Asking everyone to consider the categories the authors have identified would be reasonable, though as described by the authors, it would probably need to be tied to existing standards already in use by such repos, in order to present a low bar to usage.

Line 1002 is where the authors describe how data repositories should offer data curation services as part of DR4 (the use of controlled vocabularies). Such an idea would be very helpful to researchers, but would invariably come with a cost in time and money, and these are points that are not discussed in the manuscript.

S1

It makes sense to prefer domain-specific repositories, but researchers tend to use repositories listed in journal instructions to authors. Therefore this point would be less relevant to researchers, and more relevant to publishers. This could be part of the “call to the

community”, to evaluate if publishers state a preference for domain-specific repositories for biodiversity, where possible.

S2

It is a good idea to use domain-specific standards, but how will researchers discover the standards in their field? Perhaps suggestions relating to utilizing university libraries and other data management/stewardship teams would be useful here, as would a mention of discovery portals to standards such as AgroPortal, BioPortal, and FAIRsharing.

S3

Using controlled vocabularies in metadata standards has been suggested and even required by those standards for a long time. The authors instead state that this “is a new procedure in data submission”, however the life sciences have a long history of pairing standard formats with standard terminologies. If the authors mean that this a new thing in the biodiversity community, this should be explicitly stated.

S4

While this is very good advice, in practice researchers tend to have a “submit it and forget it” policy with the data they are required to share. Again, this could be a point where librarians/data stewards could help.

S5

Please consider dropping the “probably” in “probably grateful”; it makes a stronger statement, and I feel the authors can assume that repositories would welcome corrections to their data, especially by the authors of that data.

Clarity and Grammar

In general this manuscript is well written. Below are a list of changes that might be helpful to aid clarity and grammar.

Clarity

General comment: In a number of places in the manuscript (Abstract, “A second problem are arbitrary keywords…”; lines 67, 262-263) the authors state that keywords are insufficient for researchers’ searching needs because they need to match the researchers’ search terms *exactly*, otherwise the search will not succeed. This is only true if repositories have keyword lists that are flat and non-hierarchical. Many repositories now tie their keyword search to controlled vocabularies or even ontologies, thus allowing for a more semantically-meaningful search of their resource. As the authors use the ineffectiveness of keyword searches as a major limitation in the current system, a more in-depth look at current solutions (such as semantic searches) should be included. As an example, any repository that allows searching by Gene Ontology (GO) terms very likely be making use of such a feature. This extends to the comment about how SOLR and elastisearch can only perform exact match searches

(around line 263), and not hierarchical or fuzzy searching. This is not true; both of those search engines are useful particularly as they don't match words exactly - they can tolerate mis-spellings and even variants etc. to a degree which can be configured. Here's an example: https://www.elastic.co/blog/found-fuzzy-search.

Line 46: The authors argue that existing metadata standards need to be adapted to match users' needs with regards to dataset searching. However, in the abstract, the authors state that users' interests are "well covered" with respect to existing domain-specific standards, and state that it is a failure of uptake by large-scale repositories that is the limiting factor. These two points seem to be contradictory.

From Line 148: Complexity is described, and then the authors state that it will not be considered further in the manuscript. Why have a section devoted to it if it is irrelevant to the manuscript? Perhaps there is a way to introduce complexity with a sentence or two and then state why it is out of scope, thus removing most of this section.

Line 190: From the description of the GBIF survey in 2009, it is a user survey and *not* a query log. If I am correct in this statement, then should line 199 instead read "Apart from query logs and surveys, question corpora are another source…"? Otherwise it implies that GBIF survey 2009 is a query log.

Lines 199-240: This is a long section describing question corpora in some depth. However, question corpora are not the main focus of the manuscript. Perhaps this portion of the manuscript could be made shorter; include a summary of the types of question corpora and one or two examples of each. This is an interesting part of the manuscript, but not directly relevant.

Line 299: If MIBBI is no longer active, then in addition to the citation for MIBBI, the citation for its replacement (FAIRsharing) should also be included. The citation for FAIRsharing is https://www.nature.com/articles/s41587-019-0080-8. Additionally, while their summary of MIBBI's original purpose is correct, FAIRsharing has moved beyond "MIBBI 2.0" and is an online registry of 1000s of scientific data standards, databases and policies. FAIRsharing is concerned with making these resources discoverable to a variety of users, such as journals, researchers, librarians, funders and other policy makers.

Line 416: In the sentence, "An annotation process usually has two steps: the identification of terms based on…" I don't know what the word "terms" is referring to. In the preceding lines, the authors have just introduced the categories used throughout the rest of the manuscript, but "term" cannot mean "category" in this particular case. Additionally, How does an "artifact" relate to "phrases" and "terms"? In lines 459 and 474 this terminology is used, but I still don't really know what it means. Perhaps when this terminology is introduced, a simple example could be given.

Line 437, lines 459-60, line 515: "Multi-labeling was not allowed; only one category was permitted per artifact." Why was multi-labelling not allowed? Data curators regularly annotate datasets, journal articles and other data objects with more than one category. Why should it

not be allowed in this instance? If it were allowed, you might have received higher agreement; with only one category, annotators might have had to choose between two categories. Is this likely given the artifacts the annotators were presented with?

Line 464: The “24%” has already been linked to when 2 experts agree (in line 261), and yet is stated again in the sentence “This is the case for 24% out of all annotated artifacts”. Perhaps this sentence should be removed or modified, e.g. using “almost a quarter” or just changing the sentence.

Line 464: “Hence, the coverage of the identified information categories is still high”. The authors should clarify why coverage is considered high to avoid confusion, as 46% / 24% usage of OTHER seems like relatively low coverage / high usage of OTHER. Perhaps I’m simply misunderstanding the sentence?

Paragraph beginning at 466: This paragraph lists possible reasons why OTHER was used by annotators. Could some limitation of the instructions also be a possible reason? If the annotators didn’t understand a category, they might have been more likely to assign OTHER.

Line 471: “When adding these ratings to the QUALITY category, the results for the OTHER category decreased to 37%/13%/4%.” This is the first time the authors have used the “37%/13%/4%” style to denote 1/2/3 experts selecting a category, and as such should be explained for clarity.

Line 504: The agreement among annotators was classed as moderate in general, and excellent for some of the categories. How does this compare with other annotation exercises that were performed in the literature? I would expect that even with experts, different people annotate differently. Indeed, in Line 566 you state that the thresholds you use for agreement and frequency are not as high as in similar studies in biomedicine. Providing some context for your agreement values with respect to similar studies would be helpful.

Line 517-20: Why do these percentages show that the annotators interpreted poor agreement categories differently? An extra sentence explaining this might be useful.

Lines 521-3: In the sentence ending with “...there is no such evidence”, evidence of what? I was a little unclear what was being referred to here.

Line 523: What would be the purpose of discussion with biodiversity experts for this part? Again, a short explanation of why this would be helpful might be good.

Line 533: If PERSON always has 2 terms, then wouldn’t that naturally lead to a lack of people in one-term artifacts, rather than the reason being poor agreement between annotators?

Line 567: What “assumptions” are being referred to in this sentence?

Line 583: Metadata describe all primary data, not just scientific data. Consider removing the word “scientific” here, or some other way to make this clear.

Line 647: I think that “...this was also not to be expected” should read “...this was to be expected” (the “not” should not be present).

Line 719: OAI-ORE is mentioned here but is not present in Table 5, although others mentioned in this paragraph are (e.g. OAI-DC and QCD). Why is that?

Line 753-7: The first few sentences of this paragraph are clear, but I do not understand how they relate to the conclusion presented in the last sentence: “Hence, we aim to explore what information is available in general, descriptive metadata fields.” How does this sentence follow on from the earlier part of the paragraph please? Is it that you are only interested in conventional searches because that’s what your users use, therefore you are only looking at what is searched for in conventional general searches? I can’t figure out how that would work, as many conventional searches *will* look at indexes of all metadata fields (and not just the title description abstract or subject), even when a user types a search phrase into a generic search box. If instead, the reason that you are only looking in general fields is because that’s all you’re interested in, then just say that (e.g. say that you used title description abstract and subject because they were the easiest to pull out of the database using their equivalent dc: terms).

Line 773: What are the “identified search interests”? If you’ve already listed these, please remind us or reference the section where they are originally listed.

Line 782: The authors present the idea that generalist repositories favour generalist metadata formats, while domain-specific repositories favour domain-specific formats. This is an absolutely normal and expected way for such repositories to work, and yet it is presented almost in the style of a new finding, with the authors stating that this division of standards and repos “stands out”. Perhaps they’re trying to say something here that I have not understood? Indeed, I would be surprised if generalist repositories such as Dryad offered domain-specific formats.

The Timelines described in Lines 791 onward, and Figure 5:
This whole section and associated figure (Figure 5) are confusing to me. Why are you interested in when data standards were first introduced in the various repositories? How does this have any bearing on comparing users’ search interests to metadata fields available in repositories? This whole section and figure seems like an unnecessary diversion. If it is relevant, it needs a much better explanation as to how it relates to the rest of the manuscript. I’m not sure you need any of the timeline graphs either. *As a reader, I’m interested in how researchers’ interests and repositories’ metadata structures diverge, and where they are the same.* But I can’t tell how the timeline work integrates into that research question. Further, why would the program to create the timeline info struggle with the “result for RDF”? I would like the authors to either provide more information as to why the timelines are relevant, or perhaps remove the whole section.
If the timelines section is retained, then the legend in Figure 5 needs checking and making

clearer, and the entire section should be explained better (but keep it as short as possible please).

Line 781: “The overall statistics are presented in Table 7.” In fact, Table 7 describes the number of datasets parsed per data repository and metadata schema, as it says in the legend. Please modify the quoted sentence, as currently it seems like it is overall manuscript statistics that are described.

Line 876: Saying that OrganismTagger aborted for PANGAEA and Zenodo is not sufficient; it makes it sound like the program closed unexpectedly. If there was a serious error that prevented you from continuing, please say that. Otherwise, it seems odd to not have the numbers for these two.

Line 1010: In addition to the list provided by the authors in their repository, BioPortal provides over 800 terminologies, and over 400 Life Science terminologies are listed in FAIRsharing, which may be worth mentioning.

Lines 1067-71: This paragraph is factually incorrect. There are many vocabularies describing research methods, results and scientific data types. Searching in BioPortal results in 31 ontology matches that include the term “protocol”, 32 containing “assay”, and 34 containing “data type” hierarchies. FAIRsharing lists 14 terminologies that have a Ready status and describe experimental metadata.

Grammar

Line 4: Should there be a second “-” after “...ecosystems [1]” (to match the first dash)?

Line 72: “, to which” should be “the”

Line 149: “as heterogenous as their data.” instead of “as heterogeneous as data are.”

Line 173: In the list of items in the following sentence, it is unclear at which comma the list begins and the first half of the sentence ends: “In order to understand user behavior in search, query logs, surveys or question corpora are valid sources.”

Line 202: “...help understanding what...” is confusing; depending on what the authors mean, consider something like “Manually-generated annotations are helpful in understanding the information users are…”

Line 237: Please find a more suitable verb to replace “got” in “...got instructed on…”

Line 350: Was an exclamation mark really used as part of the example question?

Line 517: Comma is unnecessary in “Our results show,”

Line 628: Should probably read something like “Surprisingly, 52 repositories use in-house metadata schemes”, if that’s what is meant by that sentence.

Line 561: If something is “genuine” it is likely also “real”, and you may only wish to have one of these words.

Line 601: Instead of “We also examined…” perhaps something like “We classed standards as supporting Semantic Web activities if it could be expressed in RDF or OWL.”? Also, please note that there are many other formats that support ontologies and the semantic web than just these two (e.g. ttl, manchester OWL syntax, obo, JSON-LD…)

Line 744: Please remove “a” in “a documentation”

Table 8: There seems to be inconsistent row lines for each of the main rows (e.g. no line between GBIF and Dryad, but all other rows have lines after them). This is true for the entire rows: why are there lines in some places but not others?

Lines 901-3: “Overall, we determined a poor match of scholarly search interests and metadata. Metadata fields that explicitly cover information needs are just TIME and PERSON. They are used and filled in almost all inspected repositories.” I am confused about the wording here. Perhaps something like “Overall, the only metadata fields that matched researchers’ search interests closely were TIME and PERSON, which were present in almost all inspected repositories.”

Line 924: Should be “authors’” rather than “author’s”?

Line 929: Unnecessary “basically”

Line 1033: Replace “are responsible to properly document” with “are responsible for properly documenting”
