# Supplementary material for: Dataset search in biodiversity research: Do metadata in data repositories reflect scholarly information needs?
Source: PLoS One. 2021 Mar 24;16(3):e0246099. doi: 10.1371/journal.pone.0246099 (PMC7990268; doi:10.1371/journal.pone.0246099)
Supplement: Attachment — Submitted filename: response_to_reviewers.pdf [file pone.0246099.s004.pdf]

FRIEDRICH-SCHILLER-UNIVERSITÄT JENA

Department of Mathematics and Computer Science

Friedrich Schiller University Jena · Institute of Computer Science · 07743 Jena · Germany

Felicitas Löffler
Heinz Nixdorf Chair for Distributed Information Systems

Ernst-Abbe-Platz 1-4
07743 Jena

Germany

Telefon: 0049 (0) 36 41 9-46413
Telefax: 0049 (0) 36 41 9-46302
E-Mail: felicitas.loeffler@uni-jena.de

PlosONE Editorial Team

Jena, 2. October 2020

Dear Hussein Suleman,

We thank you for your kind comments and the reviewers for their thorough and constructive feedback on our manuscript following our submission for publication.

In accordance to the addressed issues, we have revised the manuscript. Please find attached to this letter a detailed reply on all issues. All line numbers in the right column refer to the manuscript with track changes. To facilitate the review, we highlighted new sentences in red color. Revised paragraphs or paragraphs moved and revised are highlighted in blue color.

We look forward to hearing from you regarding our submission. We would be glad to respond to any further questions and comments that you may have.

Kind regards,

Felicitas Löffler

PhD student and Research Associate

Friedrich Schiller University Jena, Germany

Main issues addressed by Reviewer 4 and Editor

Usage of *metadata, schemes, standard,* and *formats*	We clarified these terms at the beginning of Section B (Lines 586 - 598) and corrected the usage throughout the paper.
Methodology in Metadata Section	In accordance with the reviewer's and editor's suggestion, we revised the methodology and analyzed additional metadata standards (Section B, Lines 583 - 711).
Recommendations in Discussion Section	We agree with the reviewer that previous studies already provided recommendations for data repositories and scholars. Therefore, we revised the whole section along the FAIR Data Maturity Model as proposed by the reviewer (Section D, Lines 1013 - 1103).

Comments by Reviewer 2 and Reviewer 4

Term 'Plantas' in keyword list	We checked the keyword list again. The 5th entry in the keyword list (sorted by frequency) is indeed 'Plantas' (https://github.com/fusion-jena/QuestionsMetadataBiodiv/tree/master/data_repositories/content_analysis)
Typos and grammatical errors	We have corrected the typographical and grammatical errors.

Comments concerning Clarity by Reviewer 4

General comment: In a number of places in the manuscript (Abstract, "A second problem are arbitrary keywords…"; lines 67, 262-263) the authors state that keywords are insufficient for researchers' searching needs because they need to match the researchers' search terms exactly , otherwise the search will not succeed. This is only true if repositories have keyword lists that are flat and non-hierarchical. Many repositories now tie their keyword search to controlled vocabularies or even ontologies, thus allowing for a more	The reviewer is right to point out that *elasticsearch* provides a fuzzy-search to handle misspellings. In the manuscript, this is mentioned in Line 256. In addition, we added a paragraph with semantic search approaches in the Discussion Section (Lines 1089 - 1103).

semantically-meaningful search of their resource.	
Line 46: The authors argue that existing metadata standards need to be adapted to match users' needs with regards to dataset searching. However, in the abstract, the authors state that users' interests are "well covered" with respect to existing domain-specific standards, and state that it is a failure of uptake by large-scale repositories that is the limiting factor. These two points seem to be contradictory.	We checked the wording in Line 46 again. It refers to metadata.
From Line 148: Complexity is described, and then the authors state that it will not be considered further in the manuscript. Why have a section devoted to it if it is irrelevant to the manuscript? Perhaps there is a way to introduce complexity with a sentence or two and then state why it is out of scope, thus removing most of this section.	We added a motivation starting in Line 115.
Line 190: From the description of the GBIF survey in 2009, it is a user survey and not a query log. If I am correct in this statement, then should line 199 instead read "Apart from query logs and surveys, question corpora are another source…"? Otherwise it implies that GBIF survey 2009 is a query log	We added ``surveys" in Line 201.
Lines 199-240: This is a long section describing question corpora in some depth. However, question corpora are not the main focus of the manuscript. Perhaps this portion of the manuscript could be made shorter; include a summary of the types of question corpora and one or two examples of each.	The reviewer is right to point out that this paragraph is too long. We revised and shortened this part (Lines 209 – 229).

This is an interesting part of the manuscript, but not directly relevant.	
Line 299: If MIBBI is no longer active, then in addition to the citation for MIBBI, the citation for its replacement (FAIRsharing) should also be included. The citation for FAIRsharing is https://www.nature.com/articles/s41587-019-0080-8 . Additionally, while their summary of MIBBI's original purpose is correct, FAIRsharing has moved beyond "MIBBI 2.0" and is an online registry of 1000s of scientific data standards, databases and policies. FAIRsharing is concerned with making these resources discoverable to a variety of users, such as journals, researchers, librarians, funders and other policy makers.	We added a reference to FAIRsharing in Lines 297 – 300.
Line 416: In the sentence, "An annotation process usually has two steps: the identification of terms based on…" I don't know what the word "terms" is referring to. In the preceding lines, the authors have just introduced the categories used throughout the rest of the manuscript, but "term" cannot mean "category" in this particular case. Additionally, How does an "artifact" relate to "phrases" and "terms"? In lines 459 and 474 this terminology is used, but I still don't really know what it means. Perhaps when this terminology is introduced, a simple example could be given.	We added a direct link to our repository. The annotation guidelines are available in the repository as supplementary material (Line 418).
Line 437, lines 459-60, line 515: "Multi-labeling was not allowed; only one category was permitted per artifact." Why was multi-labelling not allowed? Data	We agree that this approach needs to be better explained. We addressed this issue in Lines 432 – 435.

curators regularly annotate datasets, journal articles and other data objects with more than one category. Why should it not be allowed in this instance? If it were allowed, you might have received higher agreement; with only one category, annotators might have had to choose between two categories. Is this likely given the artifacts the annotators were presented with?	
Line 464: The "24%" has already been linked to when 2 experts agree (in line 261), and yet is stated again in the sentence "This is the case for 24% out of all annotated artifacts". Perhaps this sentence should be removed or modified, e.g. using "almost a quarter" or just changing the sentence.	We rephrased the sentence according to the reviewer's suggestion (Line 462).
Line 464: "Hence, the coverage of the identified information categories is still high". The authors should clarify why coverage is considered high to avoid confusion, as 46% / 24% usage of OTHER seems like relatively low coverage / high usage of OTHER. Perhaps I'm simply misunderstanding the sentence?	We clarified that issue in Lines 462 – 463 and added a sentence for a better understanding.
Paragraph beginning at 466: This paragraph lists possible reasons why OTHER was used by annotators. Could some limitation of the instructions also be a possible reason? If the annotators didn't understand a category, they might have been more likely to assign OTHER.	Yes, this is indeed a plausible explanation and we added it as a fourth possible reason in Lines 477 – 478.
Line 471: "When adding these ratings to the QUALITY category, the results for the OTHER category decreased to	We added an explanation behind the percentages (Line 471).

37%/13%/4%." This is the first time the authors have used the "37%/13%/4%" style to denote 1/2/3 experts selecting a category, and as such should be explained for clarity.	
Line 504: The agreement among annotators was classed as moderate in general, and excellent for some of the categories. How does this compare with other annotation exercises that were performed in the literature? I would expect that even with experts, different people annotate differently. Indeed, in Line 566 you state that the thresholds you use for agreement and frequency are not as high as in similar studies in biomedicine. Providing some context for your agreement values with respect to similar studies would be helpful.	We reviewed other studies in the literature and indeed, our agreement values are not that low compared to similar studies. Therefore, we revised the sentence (Line 568).
Line 517-20: Why do these percentages show that the annotators interpreted poor agreement categories differently? An extra sentence explaining this might be useful	We revised the sentence starting in Line 520.
Lines 521-3: In the sentence ending with "...there is no such evidence", evidence of what? I was a little unclear what was being referred to here.	We revised the sentence to make this clearer (Lines 522 – 523).
Line 523: What would be the purpose of discussion with biodiversity experts for this part? Again, a short explanation of why this would be helpful might be good.	We address this issue in Lines 524 – 526.
Line 533: If PERSON always has 2 terms, then wouldn't that naturally lead to a lack of people in one-term artifacts, rather than	This is correct and we added more information to clarify that issue (Line 537).

the reason being poor agreement between annotators?	
Line 567: What “assumptions” are being referred to in this sentence?	We added a sentence in the methodology part of the question section (Lines 418 – 420) and we refer to it in the summary part (Line 570).
Line 583: Metadata describe all primary data, not just scientific data. Consider removing the word “scientific” here, or some other way to make this clear.	We removed the term ``scientific'' to address this issue (Line 586).
Line 647: I think that “...this was also not to be expected” should read “...this was to be expected” (the “not” should not be present).	This is correct. Therefore, we removed ``not'' in this sentence (Line 660).
Line 719: OAI-ORE is mentioned here but is not present in Table 5, although others mentioned in this paragraph are (e.g. OAI-DC and QCD). Why is that?	ORE is not listed in Table 5 as we did not further consider it. Table 5 only lists the standards with their date stamps used for the further analysis. This is clarified in Lines 767 – 768.
Line 753-7: The first few sentences of this paragraph are clear, but I do not understand how they relate to the conclusion presented in the last sentence: “Hence, we aim to explore what information is available in general, descriptive metadata fields.” How does this sentence follow on from the earlier part of the paragraph please? Is it that you are only interested in conventional searches because that’s what your users use, therefore you are only looking at what is searched for in conventional general searches? ...	We agree that the concentration on descriptive fields need a better motivation. We address this issue in Lines 796 – 798.
Line 773: What are the “identified search interests”? If you’ve already listed these, please remind us or reference the section where they are originally listed.	This is correct, and we added a reference to section A (Line 812).

Line 782: The authors present the idea that generalist repositories favour generalist metadata formats, while domain-specific repositories favour domain-specific formats. This is an absolutely normal and expected way for such repositories to work, and yet it is presented almost in the style of a new finding, with the authors stating that this division of standards and repos “stands out”. Perhaps they’re trying to say something here that I have not understood? Indeed, I would be surprised if generalist repositories such as Dryad offered domain-specific formats.	We revised the sentence (Line 822) and added a sentence for clarification in the summary part (Lines 943 – 947).
The Timelines described in Lines 791 onward, and Figure 5: This whole section and associated figure (Figure 5) are confusing to me. Why are you interested in when data standards were first introduced in the various repositories? How does this have any bearing on comparing users’ search interests to metadata fields available in repositories?	We agree that the presentation of the timelines must be justified. Therefore, we referenced it in the Discussion section (Line 1097) as they show how soon data repositories react on new standards and formats.
Line 781: “The overall statistics are presented in Table 7.” In fact, Table 7 describes the number of datasets parsed per data repository and metadata schema, as it says in the legend. Please modify the quoted sentence, as currently it seems like it is overall manuscript statistics that are described.	We addressed this issue and revised the sentence starting in Line 822.
Line 876: Saying that OrganismTagger aborted for PANGAEA and Zenodo is not sufficient; it makes it sound like the program closed unexpectedly. If there was a serious error that prevented you from continuing, please say that.	We agree that it would be good to present these numbers for all inspected files. Therefore, we ran the pipelines again and figured that a wrongly placed configuration file caused the pipeline stop. We fixed that issue and for consistency, we ran the pipelines for all files again. The numbers are presented in Table 10 and the result (annotated JSON files) are available in our GitHub repository (https://github.com/fusion-

Otherwise, it seems odd to not have the numbers for these two.	jena/QuestionsMetadataBiodiv/tree/master/data_repositories/content_analysis/NLP_analysis)<br><br>In addition, we processed all files with the BiodivTagger, a recently published text mining pipeline developed in our group.
Line 1010: In addition to the list provided by the authors in their repository, BioPortal provides over 800 terminologies , and over 400 Life Science terminologies are listed in FAIRsharing, which may be worth mentioning.	We added further terminology services to our list in the GitHub repository.
Lines 1067-71: This paragraph is factually incorrect. There are many vocabularies describing research methods, results and scientific data types. Searching in BioPortal results in 31 ontology matches that include the term “protocol” , 32 containing “assay” , and 34 containing “data type” hierarchies . FAIRsharing lists 14 terminologies that have a Ready status and describe experimental metadata.	Due to the revision of Section D, this paragraph is obsolete.
