# Supplementary material for: Dataset search in biodiversity research: Do metadata in data repositories reflect scholarly information needs?
Source: PLoS One. 2021 Mar 24;16(3):e0246099. doi: 10.1371/journal.pone.0246099 (PMC7990268; doi:10.1371/journal.pone.0246099)
Supplement: Attachment — Submitted filename: PONE-D-19-18843R3 Review.pdf [file pone.0246099.s005.pdf]

PONE-D-19-18843R3 Review

Please see my previous review for a summary of the manuscript. As this is a follow-up review, I will move directly to the discussion of the changes in this revision.

Many of the points I raised in my previous review have been successfully addressed by the authors, thank you. In particular the authors have made changes to the Discussion section regarding the FAIR Data Maturity Model resulting in a tie-in with the FAIR principles that is useful and clear. Thank you for the updates. However, there are still significant outstanding issues to resolve. These points are discussed in detail below.

Metadata Standards

In the previous review, I asked the authors to either incorporate the additional standards available via FAIRsharing and BioPortal within their evaluation, or provide reasons why they should not be incorporated. While the authors have now browsed the RDA Metadata Catalog in addition to the re3data site, the authors have not addressed this comment because they still provide too few Life Science standards in Table 2. The Life Science community has created hundreds of standards, and yet the authors present their survey of 21 standards as a comprehensive selection with a "broader perspective". They have not provided any suitable answer as to why FAIRsharing and BioPortal have not been used in the updated manuscript.

Specific questions about the metadata standards retrieval portion of the manuscript follow:

1. **Why have they used re3data to retrieve standards?**
   The authors state that **"***In re3data, we filtered for "Life Sciences" and received a list of 24 standards."* I remain puzzled why re3data, a resource which describes itself as "a global registry of research data repositories" should be used to retrieve standards.
2. **How have they used re3data to retrieve standards?**
   If I search for Life Sciences as the authors state, I get 1391 *repositories*, not 24 *standards* (the equivalent search in FAIRsharing returns 714 standards of all types with a ready status and with the Life Science tag). What search has been performed, as this should have been documented to make it reproducible? I have looked at the github repository the authors provided, but I could not find the information. The authors need to provide the methodology for how they came to retrieve *standards* from a repository of *databases*. We do not have access to their queries / URLs used to generate the original list, so we cannot confirm how the list in the supplementary material was made.
3. **How have they used the RDA Metadata Catalog to retrieve standards?**
   While the RDA Metadata Catalog does provide lists of standards for browsing, its numbers are relatively limited. Even so, there is another issue with their use of this resource. The authors stated "*From RDA, we selected all top-level standards labeled with "Science" resulting in a list of 30 standards.*" The authors have not provided their

search URL for the RDA Metadata Catalog so I cannot confirm the 30 standards they have discovered. When visiting their citation URL, I see no section heading called "Science". I see a section heading called "Life Science", but that has a different number of items than 30. The description of how they retrieved the 30 resources from the RDA Metadata Catalog is unsatisfactory, as their work cannot be reproduced. Further, the RDA Metadata Catalog is a resource that is a non-searchable, static listing of standards. Because of this no accurate count of the number of standards is visible on the site, but once again it is clear that it contains fewer records than FAIRsharing (recreating the "Science" tag as described for the RDA Metadata Catalog, FAIRsharing would retrieve 817 standards of all types with a ready status and 'Natural Science').

4. **Why did they choose to ignore the vast number of available life science standards, as listed in my previous review via FAIRsharing and BioPortal?**
   I remain confused as to why they didn't make use of FAIRsharing and BioPortal, as mentioned in my first review. The former was created expressly to provide a registry of databases, data standards and data policies. The latter provides life science ontologies, and terminologies were listed within the manuscript as suitable standards for their analysis.
   These resources seem perfectly suited to the authors' needs, and yet they chose not to use them either in the initial manuscript or after I used them as an example in my previous review. Creating a similar search to what the authors describe for re3data for FAIRsharing, the result is 714 standards of all types with a ready status and with the Life Science tag. Recreating the "Science" tag as described for RDA Metadata Catalog, FAIRsharing would retrieve 817 standards of all types with a ready status and 'Natural Science'. It is unclear why the authors, even after being told of its existence by this reviewer, have ignored this issue.
   The authors make use of a number of terminologies as well as formats, and therefore it is equally unclear as to why my recommendation of searching BioPortal was not followed. There are almost 900 terminologies in BioPortal, all of which relate to the life sciences. Clearly not all of these standards would be suitable for the authors' analysis. However, by using only re3data and RDA Metadata Catalog they have limited their initial set of standards by an order of magnitude. There may be a good reason why these two projects were left out, but no answer was provided by the authors.

Unless there is a clear reason as to why they are unsuitable for inclusion, an evaluation of the larger pool of standards via searches in FAIRsharing and BioPortal would be a required addition to the manuscript in order to determine if there truly is a large difference between researchers' search interests and the metadata fields currently available to the biodiversity community in existing repositories.

Additional comments for this section

> *We merged both lists and cleaned the final outcome according to the following criteria: The categories Other and Repository-Developed Metadata Schema have been omitted. The*

MIBBI standard is outdated and has been integrated into ISA-Tab, so we left it out, too. The same applies to the Observ-OM and CIM standard. The information on the website is deprecated and not fully available (e.g., dead links). We also omitted the "Protocol Data Element Definitions" (data elements that are required for data archival for clinical trails), all astronomy and astrophysics related standards and standards for social and behavioral studies, as these fields are out of our scope.

The above cleaning steps might be less onerous if they had used FAIRsharing, whose search query interface filters for non-deprecated resources and restricts results to the Life Sciences. Again, it's hard to understand why the authors would undertake such manual steps to clean their data when FAIRsharing has these features built in. (Please also note I believe the authors mean "trials" and not "trails" in the above text.)

> *They are ranked by the number of data repositories supporting them (obtained from re3data). We analyzed whether the standard supports semantic web formats, e.g., RDF or OWL. According to the FAIR principles [11], community standards, semantic formats, and ontologies ensure interoperability and data reuse.*

Had they used FAIRsharing they would have been able to make use of the information provided by the related links, which create associations among related standards, databases and data policies. This would allow them to see information on uptake of both the standards in question by both community and general data repositories, very helpful for their manuscript topic.

Usage of the terms *metadata, schemes, standard*, and *formats*

The authors have made the usage of the terms metadata, schemes, standard, and formats consistent across the manuscript, thank you. It is now clear that they are using the term metadata schema as defined in citation 56 (https://www.iso.org/obp/ui/#iso:std:iso:23081:-1:ed-2:v1:en).

Table 4 lists RDF as one of these metadata schemes, but it is a generic format and not a fit for a metadata schema according to the definition you're aligning with in citation 56 ("logical plan showing the relationships between metadata elements, normally through establishing rules for the use and management of metadata specifically as regards the semantics, the syntax and the optionality (obligation level) of values").Therefore RDF is not appropriate as a discussion point at all in Table 4 or in that section.

MIBBI Section

This comment relates to the following manuscript text:
The MIBBI 290 project [44] also recognized that only improved metadata allow information seekers to retrieve relevant experimental data. They propose a harmonization of minimum

information checklists in order to facilitate data reuse and to enhance data discovery across different domains. Checklist developers are advised to consider "'cross-domain' integrative activities" [44] when creating and maintaining checklists. In addition, standards are supposed to contain information on formats (syntax), vocabularies and ontologies used. Nowadays, its successor FAIRSharing [45] is an online registry offering access to numerous scientific data standards, databases and policies. FAIRSharing aims to make these resources discoverable and available to a variety of users, such as journals, researchers, librarians, funders and other policy makers.

In my previous review, I asked that the authors explain the association between the deprecated MIBBI project and FAIRsharing, as the earlier versions of FAIRsharing had their beginnings with MIBBI. My apologies, as it seems I implied that FAIRsharing could wholly be described as some kind of updated MIBBI; this is not true, as FAIRsharing is much more than that. I suggest the following change to the above text, as this more accurately reflects the purposes of both MIBBI and FAIRsharing:

"The MIBBI project[44] was the first to recognise that better metadata allows the accurate and appropriate retrieval of relevant experimental data. The MIBBI community generated a number of reporting guidelines and 'minimum information checklists' in the life sciences, revolutionising good data management and facilitating enhanced data discovery. Since then, standards have (rightly) proliferated, not just for checklists but also for terminology artefacts, identifier schema, models and formats. FAIRsharing.org [45] manually curates metadata on these standards and the relationships between them and relates this back to metadata on the repositories and knowledgebases that implement and use them. Further, FAIRsharing links both standards and databases to journal and funder data policies that recommend or endorse their use."

Discussion

Line 1045: Please note that ISA-TAB is used for far more than just "genomics" data as listed in this line. If you go to https://www.isacommons.org/, there is a list of current communities that make use of this standard, such as environmental research projects. The authors should be aware that ISA is a metadata framework that can help manage an extremely diverse set of life science, environmental and biomedical experiments that employ one or a combination of technologies. As such, they should point out that ISA-TAB may be able to accommodate an even larger part of the biodiversity community's needs than is stated in the manuscript.

As a final note, around line 1020 I am a little confused by what the authors mean by proposing to extend the FAIR Maturity Model. Do the authors mean they would like to create an extension of the Maturity Model to address their community's needs, or perhaps that they want to change that document to contain more specific recommendations? The authors need to state their goal in this section more clearly please.

Clarity

Why was DDI removed from the most recent revision in Table 2? I understand that the authors removed it from further analysis as it is primarily aimed at questionnaires/surveys, but why just drop it completely from the revised manuscript?

Throughout the paper, the authors must correct every instance of what is currently named “RDA” as “RDA Metadata Catalog” when describing this source for some of the data standards/databases, otherwise they are referring to the organisation as a whole, which is incorrect.

Figure 1: Does the example in this figure have three terms per artifact? If so, then it would be great to mention that in the caption for this figure, as later in the Results section (e.g. Table 1) there is a lot of mention of 1-, 2- and 3-term artifacts, and this figure provides a good example of what that means.

Line 436: In the following, the phrase “The latter also applied” is unclear; earlier in the manuscript (line 404) the authors state that if the phrase is fuzzy then the category NONE should apply. However, in the following text, it seems to imply that OTHER should be used for fuzzy phrases. Please could you clarify? “Should there be no proper category, the annotators were advised to select OTHER and if possible to provide an alternative category. If they did not know a term or phrase, they could decide either to look it up or to omit it. **The latter also applied** if they considered a phrase or term to be not relevant or too complicated and fuzzy.”

The labelling of the figures seems off; what is listed as Figure 3a and 3b in pages 43-44 of the manuscript seem to actually be Figures 2a and 2b. In the manuscript text, Figure 3 is meant to describe Kappa values, and yet page 45, which seems to have the bar chart that matches, is titled Figure 4. Please could the authors double check the labelling of all figures.

Figure 2: Should the bar chart legend for the dark orange have different mixed casing, e.g. “Agreement (QUALITY corrected)”? If Figure 2 will remain in the position in the manuscript it is in now, some reference to the section in which QUALITY correction is addressed should be added, as up to this point it has not been mentioned yet. Something like “The frequency of the categories and how often they were assigned to given phrases and terms, with and without QUALITY correction. See Results and Metrics for details of the QUALITY correction.” There appear to be two bar charts for figure 2, but there isn’t a caption that explains the two charts and how they differ. Should it be Figure 2a and 2b? These two bar charts also have different labels in some places, e.g. “Person & Organization” versus “Person”, and “Material & Substances” versus “Material”. Shouldn’t these be identical?

Line 531: refers to “Figure 3b depicts a more detailed picture on the individual categories”, however, in Line 456 this seems to be named Figure 2. Is this a labelling issue?

Figure 4's caption is "Fig 4. Frequency of category mentions and inter-rater agreement with QUALITY correction.", and this seems to most closely match the figure on page 55, but that is marked with "figure 2", and the legend of the graph in page 55 is confusing. Please could a longer description be provided either in the graph or in the caption, and could the labelling of the figures be checked?

Line 778: *"The header section comprises general information such as example ID of the record and a date stamp."* Shouldn't the header contain an actual ID and not an example ID?

Figure 5: All of the graphs in figure 5 are difficult to read. I understand what they are showing, but the fact that there are usually multiple lines completely overlapping means that the only way you know those different lines are present is via the legend. Somehow, these graphs need to show the different lines better - perhaps a higher resolution image, or made with different software?

A thorough examination of the 5 repositories and their metadata standards were carried out (e.g. Table 7) using NLP and their descriptive metadata fields, which I found very interesting. After reading the summary around line 939, I wonder if it would be relevant to discover which of the best performing metadata standards from Table 3 would fit with these 5 repositories? Perhaps that would lead to suggesting that those repositories, to best fit the biodiversity community's needs, should utilize these metadata standards? For example, the authors said that ISA-TAB was the best performing out of those in Table 3 - perhaps one conclusion from this would be that repositories would be better using a format such as ISA-TAB to fulfil the needs of biodiversity researchers?

Line 1067: Please provide URL to appropriate file/directory in the GitHub Repo to aid findability.

Line 1095: Not only do the EBI and NCBI provide semantic aspects to their searching, but also FAIRsharing, which you cited earlier in your manuscript, also uses semantic searches for subjects and domains. It would be useful to add FAIRsharing to the list in this sentence.

In my previous review, I had this comment: "Line 1010: In addition to the list provided by the authors in their repository, BioPortal provides over 800 terminologies, and over 400 Life Science terminologies are listed in FAIRsharing, which may be worth mentioning."
The authors replied with "We added further terminology services to our list in the GitHub repository". I have looked in the GitHub repository and can't see where this was added. Please could the authors provide the URL?

Grammar

Line 210: "Genuine user request have been" should be "Genuine user requests have been"

Line 245: "Classical retrieval models are for instance: the Boolean Model [12] where only datasets are returned that exactly match a query. It is often used in search engines in

combination with further retrieval models such as the Vector Space Model [12]. Here, the content of datasets is represented by vectors that consist of term weights." I'm not sure what is being said here. Should it be something like "Classical retrieval models include the Boolean Model [12], where datasets are only returned that exactly match a query, and the Vector Space Model [12], often used in combination with the Boolean Model. In the Vector Space Model, the content of datasets is represented by vectors that consist of term weights."?

Line 590: "A metadata schema [56] formally describe" should be "A metadata schema [56] formally describes"

Line 596: "liberal way and include both, schemata" should be "liberal way and include both schemata"

Line 682: "research interest" not "research interests"
