# Supplementary material for: Dataset search in biodiversity research: Do metadata in data repositories reflect scholarly information needs?
Source: PLoS One. 2021 Mar 24;16(3):e0246099. doi: 10.1371/journal.pone.0246099 (PMC7990268; doi:10.1371/journal.pone.0246099)
Supplement: Attachment — Submitted filename: rebuttal_letter.pdf [file pone.0246099.s006.pdf]

FRIEDRICH-SCHILLER-UNIVERSITÄT JENA

Department of Mathematics and Computer Science

Friedrich Schiller University Jena · Institute of Computer Science · 07743 Jena · Germany

Felicitas Löffler
Heinz Nixdorf Chair for Distributed Information Systems

Ernst-Abbe-Platz 1-4
07743 Jena

Germany

Telefon: 0049 (0) 36 41 9-46413
Telefax: 0049 (0) 36 41 9-46302
E-Mail: felicitas.loeffler@uni-jena.de

PlosONE Editorial Team

Jena, 22. December 2020

Dear Hussein Suleman,

We thank you and the reviewer for the comments and feedback on our manuscript following our submission for publication.

In accordance to the addressed issues, we have revised the manuscript. Please find attached to this letter a detailed reply on all issues. All line numbers in the right column refer to the manuscript with track changes. To facilitate the review, we highlighted new sentences in red color. Revised paragraphs or paragraphs moved and revised are highlighted in blue color.

We look forward to hearing from you regarding our submission. We would be glad to respond to any further questions and comments that you may have.

Kind regards,

Felicitas Löffler

PhD student and Research Associate

Friedrich Schiller University Jena, Germany

Issues addressed by Reviewer 4 and Editor

Metadata Section

Why have they used re3data to retrieve standards? I remain puzzled why re3data, a resource which describes itself as “a global registry of research data repositories” should be used to retrieve standards."	We clarified that in Lines 604 – 605.
How have they used re3data to retrieve standards? If I search for Life Sciences as the authors state, I get 1391 repositories , not 24 standards (the equivalent search in FAIRsharing returns 714 standards of all types with a ready status and with the Life Science tag). What search has been performed, as this should have been documented to make it reproducible?	We added a sentence with the URLs in Lines 605 – 608.
How have they used the RDA Metadata Catalog to retrieve standards? While the RDA Metadata Catalog does provide lists of standards for browsing, its numbers are relatively limited. Even so, there is another issue with their use of this resource. The authors stated “ From RDA, we selected all top-level standards labeled with “Science” resulting in a list of 30 standards. ” The authors have not provided their search URL for the RDA Metadata Catalog so I cannot confirm the 30 standards they have discovered…..	We provided the URLs in Lines 608 – 610 and updated the reference as we used the RDA Metadata Catalog version 2.
Why did they choose to ignore the vast number of available life science standards, as listed in my previous review via FAIRsharing and BioPortal? I remain confused as to why they didn’t make use of FAIRsharing and BioPortal, as mentioned in my first review. The former was created expressly to provide a registry of databases, data standards and data policies. The latter provides life science ontologies, and terminologies were listed within the manuscript as suitable standards for their analysis....<br><br>additional remark to the cleaning steps and criteria: "The above cleaning steps might be less onerous if they had used FAIRsharing, whose search query interface filters for non-deprecated resources and restricts results to the Life Sciences." ... "Had they used FAIRsharing they would have been able to make use of the information provided by the related	We know that numerous metadata standards have been created for different purposes and sub-domains in the Life Sciences. However, we wanted to focus on the ones that are used by large data repositories and that are supported by the global research community (Lines 604 – 605). The final list presented in Table 3 also contains the standards used in the biodiversity projects we are involved in.

links, which create associations among related standards, databases and data policies"	

Usage of the terms metadata, schemes, standard, and formats

RDF is a format, "Therefore RDF is not appropriate as a discussion point at all in Table 4 or in that section."	We added a sentence for clarification in Line (775 – 777) and updated the caption of Table 4.

MIBBI paragraph

"The MIBBI project[44] was the first to recognise that better metadata allows the accurate and appropriate retrieval of relevant experimental data. The MIBBI community generated a number of reporting guidelines and 'minimum information checklists' in the life sciences, revolutionising good data management and facilitating enhanced data discovery. Since then, standards have (rightly) proliferated, not just for checklists but also for terminology artefacts, identifier schema, models and formats. FAIRsharing.org [45] manually curates metadata on these standards and the relationships between them and relates this back to metadata on the repositories and knowledgebases that implement and use them. Further, FAIRsharing links both standards and databases to journal and funder data policies that recommend or endorse their use."	We updated the paragraph about the MIBBI project and used the second part of the reviewer's suggestion (Line 290 - 301).

Discussion

Line 1045: Please note that ISA-TAB is used for far more than just "genomics" data as listed in this line. If you go to https://www.isacommons.org/ , there is a list of current communities that make use of this standard, such as environmental research projects. The authors should be aware that ISA is a metadata framework that can help manage an extremely diverse set of life science, environmental and biomedical experiments that employ one or a combination of technologies. As such, they should point out that ISA-TAB may be able to accommodate an even larger part of the biodiversity community's needs than is stated in the manuscript.	We agree with the reviewer, that ISA-TAB can be used for different biological experiments and clarified that in Lines 1054 – 1056.

As a final note, around line 1020 I am a little confused by what the authors mean by proposing to extend the FAIR Maturity Model. Do the authors mean they would like to create an extension of the Maturity Model to address their community's needs, or perhaps that they want to change that document to contain more specific recommendations? The authors need to state their goal in this section more clearly please.	We revised the sentence accordingly (Lines 1030–1032).

Comments concerning Clarity by Reviewer 4

Why was DDI removed from the most recent revision in Table 2? I understand that the authors removed it from further analysis as it is primarily aimed at questionnaires/surveys, but why just drop it completely from the revised manuscript?	DDI is a metadata standard dedicated for the description of survey data in social and behavioral sciences. In this analysis, we only focused on Life Science related metadata standards (Line 608). Thus, we decided to omit it.
Throughout the paper, the authors must correct every instance of what is currently named “RDA” as “RDA Metadata Catalog” when describing this source for some of the data standards/databases, otherwise they are referring to the organisation as a whole, which is incorrect.	We updated the naming throughout the paper.
Figure 1: Does the example in this figure have three terms per artifact? If so, then it would be great to mention that in the caption for this figure, as later in the Results section (e.g. Table 1) there is a lot of mention of 1-, 2- and 3-term artifacts, and this figure provides a good example of what that means.	We updated the caption of Figure 1 according to the reviewer’s suggestion.
Line 436: In the following, the phrase “The latter also applied” is unclear; earlier in the manuscript (line 404) the authors state that if the phrase is fuzzy then the category NONE should apply. However, in the following text, it seems to imply that OTHER should be used for fuzzy phrases.Please could you clarify? “Should there be no proper category, the annotators were advised to select OTHER and if possible to provide an alternative category. If they did not know a term or phrase, they could decide either to look it up or to omit it. The latter also applied if they considered a phrase or term to be not relevant or too complicated and fuzzy.”	We clarified that in Line 405 and Lines 437 – 440.
Line 778: “The header section comprises general information such as example ID of the record and a	We corrected this term in Line 784.

date stamp." Shouldn't the header contain an actual ID and not an example ID?	
A thorough examination of the 5 repositories and their metadata standards were carried out (e.g. Table 7) using NLP and their descriptive metadata fields, which I found very interesting. After reading the summary around line 939, I wonder if it would be relevant to discover which of the best performing metadata standards from Table 3 would fit with these 5 repositories? Perhaps that would lead to suggesting that those repositories, to best fit the biodiversity community's needs, should utilize these metadata standards? For example, the authors said that ISA-TAB was the best performing out of those in Table 3 - perhaps one conclusion from this would be that repositories would be better using a format such as ISA-TAB to fulfil the needs of biodiversity researchers?	We discussed that suggestion but decided not to extend the summary.
Line 1067: Please provide URL to appropriate file/directory in the GitHub Repo to aid findability.<br><br>In my previous review, I had this comment: "Line 1010: In addition to the list provided by the authors in their repository, BioPortal provides over 800 terminologies , and over 400 Life Science terminologies are listed in FAIRsharing, which may be worth mentioning." The authors replied with "We added further terminology services to our list in the GitHub repository". I have looked in the GitHub repository and can't see where this was added. Please could the authors provide the URL?	We added the URL to the manuscript (Line 1077 – Line 1078).
Line 1095: Not only do the EBI and NCBI provide semantic aspects to their searching, but also FAIRsharing, which you cited earlier in your manuscript, also uses semantic searches for subjects and domains. It would be useful to add FAIRsharing to the list in this sentence.	In this paragraph, we only listed data repositories offering semantic search services. As FAIRsharing is not a data repository, it is not mentioned here. However, we listed FAIRsharing with its services in our repository (https://github.com/fusion-jena/QuestionsMetadataBiodiv/blob/master/biodivTerminologyServices.md).

Comments related to Figures

Figure 2: Should the bar chart legend for the dark orange have different mixed casing, e.g. "Agreement (QUALITY corrected)"? If Figure 2 will remain in the position in the manuscript it is in now, some	We added a legend and aligned the category naming in all Figures. All Figures are correctly placed in the manuscript. We assume that the compilation at the journal's side and the produced pdf resulted in a

reference to the section in which QUALITY correction is addressed should be added, as up to this point it has not been mentioned yet. Something like "The frequency of the categories and how often they were assigned to given phrases and terms, with and without QUALITY correction. See Results and Metrics for details of the QUALITY correction." There appear to be two bar charts for figure 2, but there isn't a caption that explains the two charts and how they differ. Should it be Figure 2a and 2b? These two bar charts also have different labels in some places, e.g. "Person & Organization" versus "Person", and "Material & Substances" versus "Material". Shouldn't these be identical?<br><br>The labelling of the figures seems off; what is listed as Figure 3a and 3b in pages 43-44 of the manuscript seem to actually be Figures 2a and 2b. In the manuscript text, Figure 3 is meant to describe Kappa values, and yet page 45, which seems to have the bar chart that matches, is titled Figure 4. Please could the authors double check the labelling of all figures.<br><br>Line 531: refers to "Figure 3b depicts a more detailed picture on the individual categories", however, in Line 456 this seems to be named Figure 2. Is this a labelling issue? Figure 4's caption is "Fig 4. Frequency of category mentions and inter-rater agreement with QUALITY correction.", and this seems to most closely match the figure on page 55, but that is marked with "figure 2", and the legend of the graph in page 55 is confusing. Please could a longer description be provided either in the graph or in the caption, and could the labelling of the figures be checked?	different ordering of the Figures. In the journal's pdf revision 3, Figure 2 is placed at the very end, because we didn't change the Figure in this revision.
Figure 5: All of the graphs in figure 5 are difficult to read. I understand what they are showing, but the fact that there are usually multiple lines completely overlapping means that the only way you know those different lines are present is via the legend. Somehow, these graphs need to show the different lines better - perhaps a higher resolution image, or made with different software?	In the journal's pdf version all Figures seem to have a reduced size. However, we checked all Figures with the tool suggested by the journal – PACE – and uploaded all Figures in this revised resolution. However, we also provide all Figures in .eps format and let the journal decide which format to take.

All grammatical and spelling errors mentioned by reviewer 4 have been corrected.
